# Wavelength and Polarization Affect Phototaxis of the Asian Citrus Psyllid

**DOI:** 10.3390/insects8030088

**Published:** 2017-08-19

**Authors:** Thomson M. Paris, Sandra A. Allan, Bradley J. Udell, Philip A. Stansly

**Affiliations:** 1 Entomology and Nematology Department, Indian River Research and Education Center, University of Florida, Fort Pierce, FL 34945, USA; thomsonparis@ufl.edu; 2 Center for Medical, Agricultural, and Veterinary Entomology (CMAVE), Agriculture Research Service (ARS), US Department of Agriculture (USDA), Gainesville, FL 32608, USA; 3 Department of Wildlife Ecology and Conservation, University of Florida, Gainesville, FL 32607, USA; bradjudell@ufl.edu; 4 Entomology and Nematology Department, University of Florida-IFAS, Southwest Citrus Research and Extension Center, Immokalee, FL 34142, USA; pstansly@ufl.edu

**Keywords:** Asian citrus psyllid, *Diaphorina citri*, phototaxis polarization, vision, behavior

## Abstract

The Asian citrus psyllid, *Diaphorina citri* Kuwayama (Hemiptera: Liviidae), is a primary pest of citrus due to its status as a vector of the citrus disease, huanglongbing. We evaluated the effects of light of specific wavelength and polarization on phototactic behavior of *D. citri* using a horizontal bioassay arena. Wavelength-associated positive phototaxis was associated with short wavelength UV (350–405 nm) targets whereas little or no responses were seen in longer wavelength targets in the visible spectrum from green to orange (500–620 nm). Distance walked towards the visual target was greater for UV/blue wavelengths (350–430 nm) than for longer wavelengths. Distances walked towards 365 nm light were greater than to white light, and distances travelled to green, yellow and orange light were similar to those in darkness. A reduced light intensity decreased responses to white and UV (365 nm) light. Polarized light was discriminated and *D. citri* travelled greater distance in response to white vertically polarized light than to horizontally polarized or unpolarized light of equal intensity. Responses to polarized 405 nm light were greater than to unpolarized light, although without an effect of polarization plane. For 500 nm light, there was no difference between responses to polarized or unpolarized light. There was no effect of age on responses to 405 nm light although 1 day old psyllids travelled faster in the presence of 500 nm green compared to 4–7 day old psyllids. Movement in response to UV and relative stasis in response to longer wavelength light is consistent with observed behaviors of settling on foliage for feeding and dispersing out of the canopy when flush needed for reproduction is scarce.

## 1. Introduction

The Asian citrus psyllid, *Diaphorina citri* Kuwayama (Hemiptera: Liviidae), is the primary vector of *Candidatus* Liberibacter asiaticus which causes the disease citrus greening or huanglongbing (HLB) [1]. This disease has had devastating effect on citrus production in the US, and resulted in a loss of US$4.54 billion in revenue and 6600 jobs in Florida alone [2]. Currently, *D. citri* is controlled in commercial citrus primarily by intensive insecticide treatments [3,4,5,6]. Combining insecticide treatments with therapeutic foliar nutrition helps to extend the productivity of HLB positive citrus trees [7]. Pesticide management of *D. citri* at low populations in citrus groves is difficult because the most cost effective method of surveillance, stem tap sampling, is not accurate at low populations [8]. While yellow sticky traps are considered more sensitive than tap sampling [8], they are not efficient for early detection of *D. citri* in citrus groves. Efforts to improve sensitivity of the standard yellow sticky trap [9] by use of enhancements such as olfactory lures based on plant and/or *D. citri* volatiles have not yielded promising results [10,11]. This may be due to visual cues such as color being the primary sensory modality for host plant location, or serving as critical components of olfactory assays [9,10,11]. More detailed insight into visually driven behaviors of *D. citri* may facilitate improvement of surveillance and management of *D. citri*.

Adult *D. citri* are known to respond to different colors. For instance, attraction of adult *D. citri* to yellow and yellow/green visual traps is well documented [9,12,13]. Additionally, attraction to UV-, green- and yellow-emitting diodes (LED) was reported by Paris et al. [14]. Further studies involving filters transmitting a broad spectrum of wavelengths indicated that *D. citri* preferred visual targets composed of both long (green, yellow) and short (UV) wavelengths to either light source alone [15]. In two choice assays, the selection of particular visual targets by *D. citri* was influenced more by the hues (colors) than intensity (brightness) [15]. Furthermore, a spectral sensitivity study determined the presence of UV, blue and green/yellow photoreceptors in *D. citri* [16]. Efficacy of metalized mulch for protection of citrus trees supports the role of UV in visual orientation by *D. citri* [17].

Three forms of visually guided behavior based on wavelength include color vision, broad band achromatic vision and wavelength selective behavior [18]. Color vision which is a complex concept that entails discrimination of wavelength independent of intensity, relies on the presence of at least two photoreceptors that differ in spectral sensitivity and utilizes a neural mechanism for discrimination of wavelengths and image [19,20,21]. Color vision is well characterized for honeybees [22,23] and some butterfly species [22,24,25]. Achromatic vision does not rely on wavelength discrimination, and includes aspects of polarized light orientation, escape phototaxis, optomotor and scanning response and orientation towards spotlight [18].

Wavelength-specific behavior, also known as wavelength-selective behavior, is defined as a stereotypic behavior that can be triggered by certain wavelengths and not by others [18,21]. While wavelength-specific behaviors may be related to color vision, unlike color vision they are dependent on light intensity, based more on discrimination of a particular color rather than contrast and mechanism of color differentiation. As color vision and wavelength-specific behavior are inextricably involved in providing guidance for visually-based behaviors, it can be difficult to discern their independent roles [18]. Wavelength-specific behavior has been reported for a range of species with the best known examples including whiteflies and butterflies. Wavelength-specific behavior in the whitefly, *Trialeurodes vaporarium* Westwood (Hemiptera: Aleyrodidae), consists of increased walking speed and more frequent take-off events in the presence of UV/violet light, whereas response to green/yellow light is lower walking speed and settling [26]. Behaviors such as feeding, drumming, egg-laying, and open space response have been observed in response to several specific wavelengths by the cabbage butterfly, *Pieris brassicae* L. (Lepidoptera: Pieridae), in association with different behaviors [24,27] and in the hawkmoth, *Macroglossum stellatarum* L. (Lepidoptera: Sphingidae), with floral choice from visual choice studies [28].

Polarization of sunlight occurs as a result of scattering from particles in the atmosphere or hydrosphere or reflection from surfaces such as vegetation and water [29]. Polarized light may be detected by specifically adapted ommatidia that are often present in specialized locations of compound eyes, typically the dorsal rim [29]. While polarization is considered monochromatic, it is associated in different species with specific receptors such UV, blue or green [30,31,32]. Behaviorally, information concerning polarization has been associated with navigational orientation or course stabilization in insects such as honey bees [33,34], ants (*Cataglyphis bicolor* F.; Hymenoptera: Formicidae) [35], crickets [36], dung beetles (*Scarabaeus nigroaeneus* Boheman; Coleoptera: Scarabaeidae) [37,38], backswimmers (*Notonecta glauca* L.; Hemiptera: Notonectidae) [39] and butterflies (*Papilio aegeus* Donovan; Lepidoptera: Papilionidae) [40].

The objective of this study was to obtain more insight into the role of visual stimuli in orientation and attraction of *D. citri* as a basis for enhancement of management or surveillance tactics. Specific objectives in this study were to examine attraction responses of *D. citri* to light of defined wavelength across the UV/visible spectrum in a walking assay and to determine if polarization, age, sex or abdominal color affected responses.

## 2. Materials and Methods

### 2.1. Insects

Psyllids were reared in a greenhouse on orange jasmine, *Murraya paniculata* L. (Rutaceae) at 29 ± 3 °C under a combination of natural light and metal halide lamps at a 16:8 L:D photoperiod. Plants were watered 3 times a week and fertilized monthly with Miracle-Gro^®^ (The Scott’s Company, Marysville, OH, USA) (N:P:K 24:8:16) (1 mL/255.7 mL water). Adult *D. citri* collected from the colony for tests represented a mixture of sexes, ages and physiological states similar to the populations present in the field. For the experiments with known age psyllids, separate rearing procedures were used. Adult psyllids were placed in a cage with a plant with flush tissue and allowed 3 days for oviposition. Then, adult psyllids were removed from the cage, eggs and nymphs were allowed to develop, all newly molted adults were collected daily and held for testing. As adults were not held individually, mating status was not controlled for this study. Different morphs of *D. citri* were characterized by abdominal colors and classified as brown/gray, orange/yellow or blue/green according to Wenninger et al. [41].

### 2.2. Bioassays

**Figure 1:**
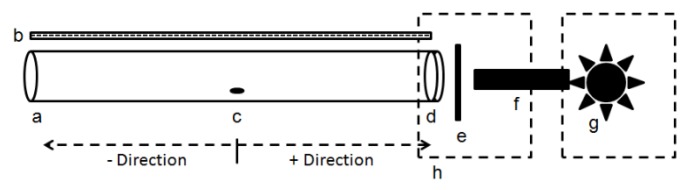
Directional bioassay arena adapted from Coombe [42]. **a**. Assay chamber consisting of a hollow cylinder (28 cm length × 2.5 cm); **b**. Transparent ruler for measuring distance traveled; **c**. Insertion hole (14 cm from each end) for psyllid introduction. In relation to the release hole, if psyllids moved towards the light, the response was considered positive. If movement was away from the light, the response was considered negative; **d**. Narrow bandpass filter placed within the end of the tube; **e**. Holder for neutral density or polarization filters; **f**. Quartz light guide, **g**. Light source; **h**. Blackout enclosures.

A testing arena modeled after Coombe [42] was constructed to evaluate the effect of different colored visual targets on the innate preference of direction and distance walked (Figure 1). The arena for this assay consisted of a clear acrylic cylinder (28 cm long and 2.5 cm internal diameter) with a release hole (0.5 cm) in the floor of the tube that was 14 cm from each end. This hole was used for insertion of test psyllids into the arena. A clear ruler was placed along the length of the arena to evaluate direction of movement and distance of the final position at the conclusion of the assay. Direction was considered positive if the psyllid had moved from the insertion hole towards the light source and negative if the psyllid moved away from the light source. At one end of the cylinder, a narrow bandpass interference filter (2.5 cm diam., Edmund Optics, Barrington, NJ) providing spectrally defined light was mounted onto a stand resting flush against the end of the cylinder. A tungsten lamp (15 w) (Carl Zeiss, Göttingen,- Germany) was used to illuminate the visual targets. A quartz light guide (Edmund Optics, Barrington, NJ, USA) transmitted light from the light source to the filters. Black out enclosures around the end of the light guide and filters were used to remove stray light. All experiments were conducted in complete darkness. Intensity of the visual targets was adjusted using neutral density filters (Thor Labs, Newton, NJ, USA) placed between the light source and the narrow bandpass filter. Neutral density filters reduce overall light transmission with no bias to wavelength and can be used to equilibrate light intensity for controls used in comparison to colored filters. The intensity of light from the visual target was measured using a concave grating spectrometer (UV-VIS BLACK-Comet; StellaNet, Tampa, FL, USA).

Psyllids were collected from rearing cages using an aspirator, placed into vials and adapted in complete darkness for at least 15 min. An individual psyllid was inserted using a paintbrush into the release hole for each assay. Psyllids were transferred to and from the arena under low intensity red light (2.94 W m^−2^ s^−1^) (624 nm). Each assay was conducted for 1 min; direction and distance of movement were noted. Movement was considered positive if the psyllids walked towards the visual target from the release point or negative if the psyllids walked away from the visual target. Time was noted if a psyllid walked to either end of the arena before a minute lapsed. The maximum distance from the insertion hole that a psyllid could travel in 1 min was 14 cm in either direction. If a test was conducted with no psyllids reaching the end before 1 min, then all data were calculated as distance travelled in 1 min. If a test included psyllids that reached the end of the arena before 1 min, all data were converted to speed (cm/min). Each psyllid was tested only once and held individually after the test so that sex and color morph could be determined. All experiments were continued until a minimum of 35 adult psyllids (a combination of males and females) were tested. All bioassays were conducted between 10:00 and 18:00 h.

### 2.3. Effect of Wavelength

Comparisons of assay responses between wavelengths were made using narrow bandwidth (10 nm) interference filters that corresponded to a maximum transmission of 350 (UV), 365 (UV), 380 (UV), 405 (violet), 430 (indigo), 500 (green), 550 (green), 580 (yellow) and 620 nm (orange/red). Final direction of movement and distance of each psyllid was also obtained. Psyllid response to white light and light at 365 nm at different intensities was obtained to better characterize the effect of light intensity on response in the assay arena. Comparisons were made between unfiltered white light or from the 365 nm filter, and light mitigated with neutral density filters over a range of optical densities (OD) (0.1–4.0).

### 2.4. Effect of Polarization

The effect of polarization was evaluated using the same assay setup except that linear polarization filters (Edmund Optics, Barrington, NJ, USA) and neutral density filters were placed in the filter holder between the light source and interference filter (Figure 1). The direction of polarization was determined a priori to be 0° (horizontal) or rotated 90° (vertical). Polarization effects were evaluated using white light as well as using light transmitted through 405 and 500 nm filters. Neutral density filters (0.4 OD) were added to unpolarized light to compensate for loss of intensity due to the polarization filter.

### 2.5. Effect of Age

Bioassays were conducted to compare behavioral responses of psyllids that were 1 or 4–7 days old to visual targets. Bioassays were conducted as above, except that only short wavelength (405 nm) and medium wavelength (500 nm) filters were used.

### 2.6. Statistical Analysis

The direction of psyllid movement was calculated as positive or negative phototaxis and compared to random directional movement which would have been 50% positive phototaxis. Χ^2^ comparisons were made using R [43] and all other comparisons were made with SigmaStat (SysStat Software, San Jose, CA, USA). Effects of color morph, sex and responses to different intensity or color were compared by ANOVA or Kruskal–Wallis on ranks if data passed or failed, respectively, the Shapiro–Wilk test for normality. Means comparisons were conducted by Fisher LSD or Tukey’s test on ranks. Data for comparisons of two means were tested for equality of variance and means compared by Student’s *t*-test or Welch’s t test if variance was equal or not, respectively.

## 3. Results

### 3.1. Effect of Wavelength

No effect of abdominal color morph (*F* = 0.06, df = 2,175, *p* = 0.94) or sex (*F* = 1.08; df = 1,176; *p* = 0.30) on psyllid response was observed for any study so data were pooled for color morph and sex. Significantly positive phototactic responses towards the light source were obtained in response to short wavelength light, i.e., 350, 365, 380 and 405 nm, with the strongest phototactic responses in the presence of 350 and 365 nm light (Table 1). Responses to the 500, 550, 580 and 620 nm filter did not differ from random orientation (50%) and were about half of those to the 350 and 365 nm filters. Although light intensity increased with wavelength, greatest responses were associated with the lowest light levels which is the opposite of what was expected. This result indicated that positive phototaxis was in response to wavelength rather than intensity.

**Figure 2:**
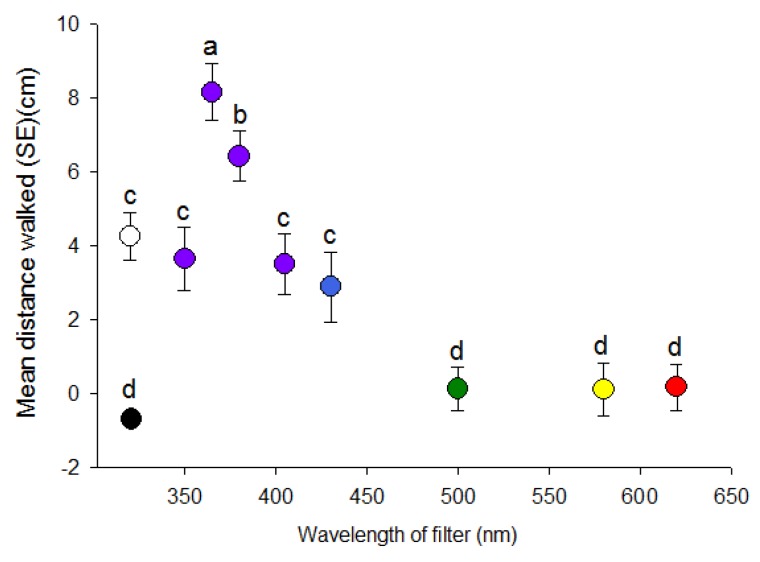
Distance (mean ± SE) (cm) walked by individual *D. citri* in response to light through narrow bandpass filters of different wavelength maxima (colored circles) as well to white light (clear circle) and complete darkness (black circle). Different letters indicate significant differences between means (paired *t*-test, *p* < 0.05).

Distances walked by psyllids differed significantly among wavelengths of visual targets in concert with direction (H = 96.03, df = 9, *p* < 0.001) (Figure 2). Distance walked towards the 365 nm visual target was about 3-fold greater than to the 350 and 405 nm targets. Psyllids only walked an average of 0.10 cm towards the 500–620 nm targets with no significant effect of wavelength. All UV/blue wavelengths (i.e., 350, 365, 380, 405 and 430 nm) elicited significant positive phototaxis, whereas the longer wavelengths (500–620 nm) were not different from complete darkness despite higher intensity of the longer wavelengths. Responses to white light were similar to 350, 380, 405 and 430 nm (Figure 2).

**Figure 3:**
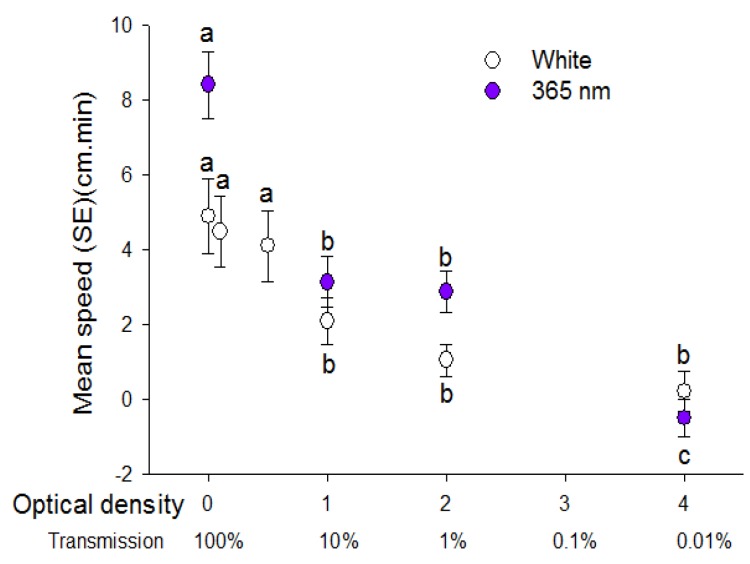
Speed (mean ± SE) (cm/min) walked by individual *D. citri* in response to white light and 365 nm light reduced in intensity with additional filters of different optical density. Transmission of light through filters is presented below optical density values. Different letters indicate significant differences between means of each type of light (paired *t*-test, *p* < 0.05).

Walking speed of psyllids towards light sources was significantly diminished in response to white and 365 nm light intensity reduced to 10 and 1% using optical density filters (H = 12.98, df = 3, *p* = 0.005; H = 41.18, df = 3, *p* < 0.001, respectively) (Figure 3). There was no difference in positive phototactic response between unfiltered white light and light reduced to 79.1% and 31.6% by use of the 0.1 and 0.5 OD filters respectively. Reduction of white light to 10, 1 and 0.01% by use of the 1, 2 and 4 OD filters respectively also reduced phototactic response compared to unfiltered white light.

### 3.2. Effect of Polarization

**Figure 4:**
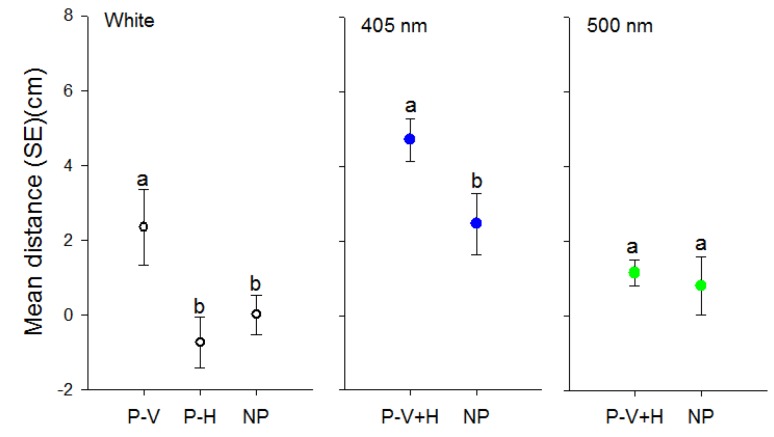
Effect of polarization of visual targets of different wavelengths on distance (mean ± SE) (cm) walked by individual *D. citri* towards targets. Targets consisted of white light or light transmitted through 405 or 500 nm filters. P-V designates use of a vertical polarization filter, P-H a horizontal polarization filter and P-V+H represents averaged data from both vertical and horizontal filters due to a lack of difference between the two orientations. NP designates non-polarized light balanced in intensity through use of neutral density filters. Different letters indicate significant differences between means within each color comparison (*p* < 0.05).

The effect of polarization of white light significantly affected walking distances of psyllids (*F* = 4.40, df = 2, 103, *p* = 0.01). Psyllids only travelled towards vertically oriented white light in contrast to horizontally polarized or unpolarized light (Figure 4). However, the orientation of the polarization filter (0° or 90°) did not affect the response of *D. citri* towards 405 nm (*t* = 0.81, df = 68, *p* = 0.208) or 500 nm light (*t* = −1.08, df = 68, *p* = 0.28). Therefore data for the two orientations of polarization filters were combined for each wavelength. Psyllids travelled greater distances towards the polarized short wavelength (405 nm) target compared to the unpolarized target (*t* = −2.63, df = 70, *p* = 0.01) (Figure 4). In contrast, there was no difference in distance travelled towards the 500 nm target in the absence or presence of polarizing filters (*t* = −0.36, df = 43, *p* = 0.72) (Figure 4).

### 3.3 Effect of Age

**Figure 5:**
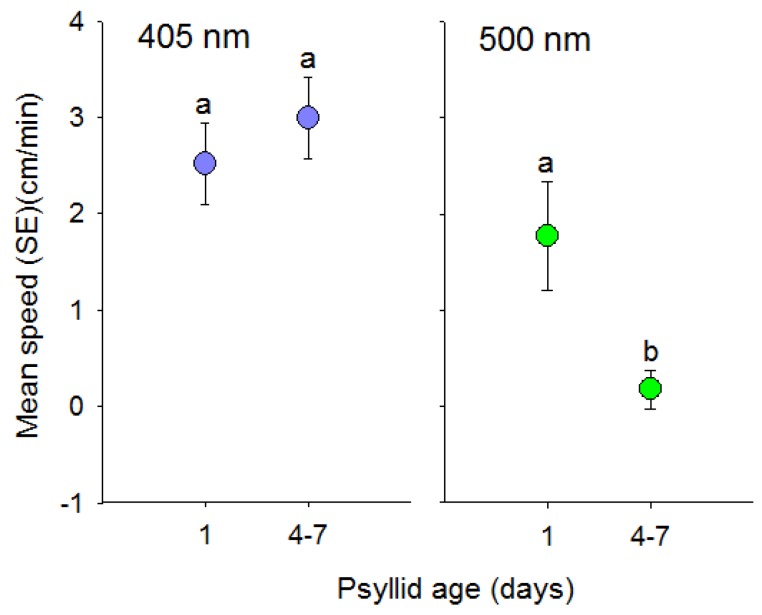
Comparison of age on the speed (mean ± SE) (cm) that individual *D. citri* walked towards visual targets that consisted of short (UV, 405 nm) or long wavelength (green, 500 nm) light. Adult psyllids were either 1 or 4–7 days old. Different letters indicate significant differences between means (*p* < 0.05) within each color comparison.

Speed of positive phototactic movement in response to short wavelength (405 nm) visual targets did not differ with age of *D. citri* (*t* = −0.58, df = 74, *p* = 0.56) (Figure 5). In contrast, day old psyllids walked farther than 4 to 7 day old psyllids in response to long wavelength (500 nm) light (*t* = 2.02, df = 92, *p* = 0.05).

## 4. Discussion

The horizontal bioassay demonstrated wavelength-specific behavior with *D. citri* displaying positive phototactic behavior in the presence of UV light (350–405 nm) but not under longer wavelengths (500–620 nm). This result is consistent with a prior study on *D. citri* spectral sensitivity that provided evidence of UV, blue and green photoreceptors [16].

Like many other herbaceous insects feeding on young flush, *D. citri* is attracted by green and yellow targets [9,12,13,14,15,44]. However, we found that *D. citri* did not move either toward or away when illuminated in light in defined wavelengths including from green, yellow and orange (500–620 nm). Dominance of these wavelengths may mimic conditions within a tree canopy consisting of green light sifting through foliage, and where olfactory and auditory cues are used to locate flush and mates [41,44]. In contrast, short wavelengths of ultraviolet through violet (350–405 nm) were attractive, especially when combined with green or yellow light [15]. Behavioral responses to UV light are generally associated with flight and dispersal [18] which would assist *D. citri* in location of new plants for infestation. Attraction to UV would lead the insects out of the canopy when flight becomes necessary due to insufficient flush or an inappropriate host. Our results provide clear evidence of wavelength-dependent behavior and are similar to those for *T. vaporariorum* which tended to fly when exposed to short wavelengths (405 nm) and settle when exposed to longer wavelengths (500 nm) [26,42].

Sensitivity of *D. citri* to UV light has been previously reported with both attraction and repellency being documented. Attraction to light-emitting LEDs in laboratory assays was reported by Paris et al. [15] with similar levels of response to UV, yellow and green LEDs. Of the range of UV-emitting LEDs tested, greatest responses were to LEDs emitting 375 nm [14]. Additionally, Paris et al. [14] reported that the addition of UV light to green or yellow enhanced attraction to targets comprised of transmitted light. In contrast, use of reflective mulch that reflects high levels of UV light was effective in reducing numbers of *D. citri* on young citrus saplings and resulted in lower levels of huanglongbing [17]. Presumably the differences between attraction and repellency are related to factors such as levels of UV light or the location of stimulating receptors in different regions of the eye. Mulch reflecting UV light produces UV reflection from below a psyllid in flight, whereas UV-emitting sources or reflection from vegetation would be in front or from above the eye.

Polarization sensitivity has been associated with UV, blue or green photoreceptors in insects [45]. However, association of polarization sensitivity with photoreceptor types in phytophagous Hemiptera has not previously been documented. In this study, we provide evidence of strong positive phototactic responses to both white and UV/violet (405 nm) polarized light. Responsiveness to polarized light in the UV region appears to be characteristic of day-active insects [46]. Use of UV receptors as detectors for sky light polarization is effective, particularly under cloudy conditions when the degree of linear polarization is highest in the UV spectrum [45,47].

Polarization of short wavelength light increased walking distance of *D. citri* towards a visual target. Direction (plane) of polarization influenced attraction to white light but not to 405 or 500 nm light indicating that neither wavelength accounted for discrimination between polarization planes seen with white light. Similarly, responses were greater to polarized compared to unpolarized 405 nm light although there was no effect of polarization direction. Clearly, *D. citri* discriminate between polarized and unpolarized light with consequent effects on phototactic behavior. This is the first study documenting *D. citri* response to polarized light. In nature, polarized light comprised of short wavelengths is primarily visible in the sky as a result of light interacting with the atmosphere [22,48]. Positive phototaxis towards polarized UV may indicate its use by *D. citri* to guide dispersal. Polarized light has been documented to aid in navigation of insects returning to a home base [49,50]. Although light reflected from plants is also polarized, attraction towards the green visual target (500 nm) was not altered by polarization. Citrus plants such as *Citrus sinensis* ‘Hamlin’ and ‘Valencia’ (Rutaceae) infected with huanglongbing reflect polarized light at different planes from uninfected citrus plants [51]. Also, polarization is greatest from dark plant surfaces with shiny leaves (such as mature leaves) which reflect higher levels of polarized light with an e-vector perpendicular to the leaf surface [46]. Our results using transmitted light suggest that polarization from host plant surface is not a factor in *D. citri* phototactic behavior, although evaluations of reflected light from healthy and huanglongbing-infected plants would be useful to detect a possible role of polarization in *D. citri* host selection.

Polarization sensitivity is more strongly associated with the dorsal rim area of compound eyes [52] which would be strongly stimulated during flight with illumination from above. Similar to Kinoshita and Arikawa [53], our studies were conducted with the stimuli in front of the insects to evaluate phototactic behavior of walking insects. Our results do not preclude that additional polarization sensitivity may exist in response to stronger stimulation of the dorsal rim area of the eyes where polarization is generally detected. Polarization effects stimulating the anterior portion of the eye may be related to directional navigation, presumably for host plant location. Further research with flight assays could clarify this point.

Intensity did not affect mean walking distance of *D. citri* until 90% transmission was blocked. Intensity changes where 90% or greater light was blocked caused *D. citri* response to visual targets to diminish. It is apparent that there is a critical threshold for *D. citri* to respond to visual targets. Once that threshold of intensity of the visual target is met, the variation in intensity is not an issue. Illumination of host plants affects host plant selection by *D. citri* with adults reported to select illuminated host plants compared to non-illuminated plants [46]. Olfactory experiments involving *D. citri* require some light intensity in order to obtain behavioral responses from *D. citri* [9,10,11]. Preference for host plants by virgin female carrot psyllids, *Trioza apicalis*, was altered by increased light intensity with more females selecting illuminated non-host plants [54].

Age had an impact on visual responses of *D. citri*. One day old adults showed greater positive phototaxis towards long wavelength targets (500 nm) compared to 4–7 day old adults. The strong drive by newly emerged adult *D. citri* to move towards long wavelength targets (500 nm) constitutes a primary behavioral strategy for finding feeding sites. Sexual maturity of *D. citri* occurs 2–3 days after emergence [55]. There were no differences between the responses of male or female psyllids to either short or long wavelengths in either age group. The increased attraction of UV light by older psyllids may indicate that older psyllids are more driven to disperse to find better host plants for feeding, oviposition and mating [55]. In a study on carrot psyllids, *Trioza apicalis* Foerester (Hemiptera: Triozidae) increased light intensity altered preference for host plants by virgin females with a change from host plants to non-host plants [54]. Shifts in host plant location by *D. citri* have been reported with more frequently successful plant location when host plants were illuminated compared to non-illuminated plants [46]. Both sexes appeared to respond similarly to visual stimuli, likely indicating that for the specific behavior evaluated in these assays, there was no bias in response. This does not preclude the potential for differences between sexes in response to visual stimuli pertaining to other behaviors.

Three color morphs (gray/brown, green/blue and orange/yellow) have been characterized from populations of *D. citri* [56,57] with variations in fitness, body mass, fecundity [56] and insecticide susceptibility between morphs [58]. A recent study by Martini et al. [59] reported longer duration flights by green/blue morphs compared to gray/brown morphs, presumably related to propensity for dispersal. In our study, there were no differences between color morphs as measured in the walking movement of *D. citri* under a range of wavelengths. These results may reflect equal propensity for localized movement for feeding within the tree canopy.

## 5. Conclusions

Wavelength-specific behavior was demonstrated by a strong walking response towards UV, particularly 365 nm; little or no walking responses were observed towards green (500 nm) or yellow (580 nm) light, which were similar to psyllid responses in darkness. Both of the latter colors are known to elicit landing and possibly flight responses by *D. citri* as characterized using LED lights [14] and yellow or yellow/green sticky traps [9,10,12,13]. Polarization of UV visual targets induces *D. citri* to increase the distance walked towards a target, which may indicate use of polarized UV light to orient during dispersal. Furthermore, there were differences between ages on mean walking distance when *D. citri* are exposed to long wavelength visual targets corresponding to dispersal behavior in natural settings. The results of this study may be useful in providing insights into the ecology of *D. citri*.

## Figures and Tables

**Table 1:** Positive phototactic responses of *D. citri* in the presence of light of specific wavelengths. Insects were scored as positive if they moved from the central insertion point towards the visual target and as negative if they moved away from the target. N = 35. Data were analyzed by Χ^2^ analysis (n = 35). Data were significant if phototactic response was greater or less than the expected value of 50% (*p* < 0.05).

Wavelength (nm)	% Positive Phototaxis	*p* Value	Light Intensity (W/cm^2^/sec)
350	85.7	<0.001	0.04
365	94.3	<0.001	0.06
380	80.0	<0.01	0.07
405	74.3	<0.01	0.08
430	60.0	0.233	0.09
500	45.7	0.612	0.12
550	54.3	0.612	0.13
580	48.6	0.866	0.12
620	45.7	0.612	0.01
